# The College Man in Dentistry

**Published:** 1900-11

**Authors:** Harry L. Grant

**Affiliations:** Providence, R. I.


### THE COLLEGE MAN IN DENTISTRY.

BY HARRY L. GRANT, D.M.D., PROVIDENCE, R. I.

All acknowledge that it is the duty of every man to become as fully developed mentally and physically as his capacity will allow. On this point the old and well-tried saying, "Heaven helps him who helps himself," is particularly applicable. No college educates the man; it simply makes it possible for him to educate himself. The development comes from within and not from without. That a man may become fully educated without the college training is equally true, as many examples show; but when one considers "the greatest good to the greatest number," the *summum bonum,* it is acknowledged, without exception, that, other things being equal, the man of average mental capacity is better fitted for life's work, whatever that may be, if first he have such training as is received in the average college course of to-day. To be sure, all who take such a course do not become so mentally trained, but the average man, of average ability, with an average ambition, never regrets the time and money required for the undertaking, and, what is more important, his work in life, whether it be in business or in a profession, will certainly show marks of the broad and deep foundation upon which his "specialty" has been erected.

The amount of knowledge an individual takes with him from college is very little. What, then, does he accomplish in the four years' course? He learns better how to use his brain, how to study, how to systematize his work, how to concentrate his powers, how to persevere. He has a broader, better view of life, he is freer from jealousies, he is less conceited, and has a more charitable view towards his colaborers.

Few, if any, ever have cause to regret a college training. Many who have attained great successes in life regret this lack. They feel that the foundation is weak. The superstructure has been built with the utmost care, but upon an insufficient foundation.

Who is better able to judge than those who have tried it? What better argument is there than the fact that the father who is college-bred, almost without exception, sees to it that the son has the same advantages, whatever calling the son may follow.

Once a college education was regarded superfluous unless a man was to enter a profession; that is, unless he was to become a teacher, a clergyman, a lawyer, or a physician. At the present time the larger number enter business, every branch of business, including mechanical pursuits. Many take technical courses, and hold their own with men who have spent all their time in technical or manual training.

Is the man who enters the shop at a tender age a better machinist than he who has had a liberal education and allows his mechanical ability to develop as he develops his whole mental power and learns how to use his brain? Cannot the man of liberal training the better direct his power, the better acquire manual dexterity, than the other? It may take longer, but will not his attainment be equal to or greater than that of the other?

Many college men become physicians; few become dentists. That there are very many finely educated men in the dental profession one can readily see by reading the reports of the various society meetings. There are as many great scholars in the dental world as there are in any of the other scientific pursuits, but they are few as compared with those of the rank and file. Does more mental training take away the power to develop manual dexterity? The spark of mechanical ability is born with the child. Will he develop it the less if he has a liberal training?

It is claimed that "The advance of the preliminary entrance standard to the very highest degree possible at the present time—the diploma of a high school, unless that be a high school of manual training—*promises to lower the standard of dental attainment.*" The writer does not wish to affirm or deny this statement, but with his present limited knowledge it does seem as if the rank and file of the dental profession would show as much manual dexterity, would be more professional in their relations with one another, would show fewer cases of commercialism, fewer cases of moral depravity, if there were more college men among them. And in pursuance of the same thought, to the writer it seems desirable that some schools—not all—should not stop with the high-school diploma, but year by year raise the standard to a college degree.

If it is desirable that the physician, the lawyer, and the clergyman should have a college training, it certainly is just as desirable that the dentist should have the same. When a college man reads in a dental journal that more mental capacity means less "dental attainment," it sends a certain twinge through his nervous system, with a desire to hear more from college men in the dental profession who have better opportunities for observation than he. He recalls several college class-mates, with marked manual dexterity, who have since attained greater distinction than their fellow-workers, because of the skill coupled with larger mental capacity. The difference between the laborer and the man who plans and carries out the work is one of mental capacity. Manual dexterity requires mental capacity. Every one cannot have the same amount of brain-power in the ends of his fingers, and to the writer's mind that power cannot be lessened in the least by requiring a high standard for entrance to the dental school. The partial elective system of college allows one to follow out the work for which one is best fitted, but does not allow him to become one-sided, as he might by following one line of work only.

Is there not something of the same difference to-day between the man of liberal education in dentistry and the one without, that existed years ago between the graduate of a dental school and the one who studied in an office only? Is not the one a step in advance of the other?

The dental profession cannot have too many liberal, fair-minded men. The man of college training or *its equivalent* has greater mental capacity, better judgment, better self-control, better power of concentration and application, more fraternal feeling, and is on a higher plane than the same individual would be without such training. Are not these qualities desirable in the dental profession at a time when we hear so much and see so much of so-called "quackery," "commercialism," enmity, and jealousy? Laws are powerless to overcome such evils. It can be done only by the education of the individual, and in what manner can that be attained except by requiring a high preliminary education for entrance into the dental profession?

